# Prognostic impact of CD34 and SMA in cancer‐associated fibroblasts in stage I–III NSCLC

**DOI:** 10.1111/1759-7714.13248

**Published:** 2019-11-24

**Authors:** Arik Bernard Schulze, Lars Henning Schmidt, Birthe Heitkötter, Sebastian Huss, Michael Mohr, Alessandro Marra, Ludger Hillejan, Dennis Görlich, Peter J. Barth, Jan Rehkämper, Georg Evers

**Affiliations:** ^1^ Department of Medicine A, Hematology, Oncology and Pulmonary Medicine University Hospital Muenster Muenster Germany; ^2^ Gerhard Domagk Institute of Pathology University Hospital Muenster Muenster Germany; ^3^ Department of Thoracic Surgery Rems‐Murr‐Klinikum Winnenden Winnenden Germany; ^4^ Department of Thoracic Surgery Niels‐Stensen‐Kliniken Ostercappeln Ostercappeln Germany; ^5^ Institute of Biostatistics and Clinical Research, Westfaelische Wilhelms‐University Muenster Muenster Germany; ^6^ Institute of Pathology, University of Cologne Cologne Germany

**Keywords:** Cancer associated fibroblast, CD34, EMT, NSCLC, SMA

## Abstract

**Background:**

Epithelial‐to‐mesenchymal transition (EMT) is a crucial step in lung cancer pathogenesis. Among others, cancer‐associated fibroblasts (CAFs) are reported to regulate this process.

**Objectives:**

To investigate the prognostic and clinical impact, we analyzed CD34+ and SMA+ CAFs in non‐small cell lung cancer (NSCLC).

**Methods:**

Retrospectively, immunohistochemistry was performed to study stromal protein expression of both CD34 and SMA in 304 NSCLC patients with pTNM stage I‐III disease. All tissue samples were embedded on tissue microarrays (TMAs).

**Results:**

Our analysis revealed an association for CD34+ CAFs with G1/2 tumors and adenocarcinoma histology. Moreover CD34+ CAFs were identified as an independent prognostic factor (both for progression free survival [PFS] and overall survival [OS] in stage I‐III NSCLC). Besides, SMA+ expression correlated with higher pTNM‐tumor stages and lymphatic spread (pN stage). In turn, SMA‐negativity was associated with improved PFS, but no prognostic impact was found on OS. Of interest, neither CD34+ CAFs nor SMA+ CAFs were associated with the primary tumor size, localization and depth of infiltration (pT stage).

**Conclusions:**

CD34 was identified as an independent prognostic marker in pTNM stage I‐III NSCLC. Moreover, loss of CD34+ CAFs might influence the dedifferentiation of the NSCLC tumor from its cell origin. Finally, SMA+ CAFs are more prevalent in NSCLC tumors of higher stages and lymphonodal positive NSCLC.

**Key points:**

- Expression of CD34 on cancer associated fibroblasts (CAFs) is an independent prognostic factor in stage I‐III NSCLC.
- SMA+ cancer associated fibroblasts are associated with higher tumor stages in NSCLC and might contribute to tumor progression in NSCLC.

## Introduction

Today, lung cancer is still one of the most common and most lethal cancer types worldwide with rising incidence rates.^1^, ^2^ With a focus on histology, two major subtypes must be distinguished from each other: While non‐small cell lung cancer (NSCLC) is the more frequent subtype, small cell lung cancer (SCLC) is considered to be the most aggressive.^1^, ^3^ NSCLC comprises over 80% of the cases with a rising incidence of adenocarcinoma (ADC) and decreasing levels of squamous cell (SCC) and large cell carcinoma (LCC).^4^ Therapeutically, besides cytotoxic chemotherapies^5^ and molecular targeted therapies (eg, EGFR‐,^6^ ALK‐ and ROS‐mutation antagonists^7^ or anti‐angiogenic antibodies against VEGF^8^, ^9^), immune‐checkpoint‐inhibition^10^, ^11^, ^12^, ^13^, ^14^, ^15^, ^16^ has recently augmented our therapeutic armamentarium for locally advanced and metastatic NSCLC.

When addressing stromal tumor tissue, three relevant factors need further consideration. Besides tumor infiltrating cells, such as macrophages, T‐lymphocytes, natural killer cells and dendritic cells,^17^ tumor microenvironment predominantly comprises tumor induced angiogenesis^18^ as well as cancer‐associated fibrocytes and fibroblasts (CAFs).^19^ Antivascular therapies and tumor infiltrating cytotoxic T‐lymphocytes have already been reported to increase the therapeutic efficacy against lung cancer. However, analysis of stroma in NSCLC is challenging due to its cell‐heterogeneity and complex cell‐cell interactions.^20^, ^21^, ^22^

Metastatic conversion, a crucial step in cancer progression, is driven by epithelial‐to‐mesenchymal transition (EMT). EMT results in loss of cell polarization, reduction of cell‐cell‐junctions and in turn gain of motility and invasiveness.^23^ Upon activation via transforming growth factor‐β (TGFβ), receptor tyrosine kinases (RTK) or the Wnt‐pathway, Snail and Zinc‐finger proteins downregulate the transcription of essentially involved proteins, such as E‐cadherin.^23^ With a focus on pathogenesis, in vitro TGFβ was able to transform clusters of differentiation 34 (CD34)+ fibrocytes into alpha‐smooth muscle actin (SMA)+ fibrocytes.^24^ Moreover, in contrast to regular fibroblasts, in vitro cell culture analyses of SMA+ CAFs revealed SMA+ CAFs induced EMT in different NSCLC cell lines.^25^ In other cancer types, such as pancreatic cancer,^26^ cervical cancer,^27^, ^28^ oral squamous cell cancer^29^ and cholangiocarcinoma^30^ as well as colorectal cancer^31^ and thyroid papillary carcinoma^32^ stromal CD34+ and SMA+ CAFs could be associated with distinct malignant features such as lymphonodal metastasis. So far, these analyses have not been performed for NSCLC.

CD34 was first detected by Civin *et al*. in 1984 and was subsequently characterized as a hematopoietic progenitor cell marker.^33^ Recently, increased expression of CD34 and CD31 has been reported on vessel endothelium and endothelial progenitor cells, especially in tumor vascularization.^34^ Here, monoclonal antibodies against CD34 and CD31 have been used to analyze the microvessel density (MVD) in central and peripheral regions of NSCLC tissue. Among others, Ushijima *et al*. considered high peripheral MVD to correlate with more advanced tumor stages and a higher probability of nodal metastasis.^35^ Nevertheless, CD34+ fibrocytes and fibroblasts have been described in wound healing^36^ and, focusing on the respiratory system, especially in fibrotic remodeling in asthma,^37^ chronic obstructive airway disease, emphysema^38^ and possibly in idiopathic pulmonary fibrosis.^39^

SMA is a marker of CAFs^40^, ^41^ and promotes exosomal cancer proliferation in NSCLC,^42^ and was first described by Gabbiani *et al*. in 1975.^43^ Since then, SMA has been used to analyze stromal features in different tumor entities, such as pancreas carcinoma,^26^ ductal adenocarcinoma of the mammary gland,^44^ cervical cancer,^28^ head‐ and neck squamous cell carcinoma,^45^ colon carcinoma,^31^ cholangiocarcinoma^30^ and squamous cell carcinoma of the lung.^46^ In conclusion, SMA+ CAFs can more frequently be found in malignant tissue than in benign alterations of the respective tissue origin and, in addition, SMA+ CAFs are more abundant in the primary tumor and in macrometastases compared to micrometastases and tumor cell‐poor lymphatic vessel spread.^30^, ^46^

Against this background, we investigated the impact of CAFs in NSCLC. We focused on CD34 and SMA as immunohistochemical biomarkers to evaluate the clinical and prognostic influence on stromal cells in stage I‐III NSCLC.

## Methods

### Study population

Retrospectively, we studied the expression of CD34 and SMA in patients suffering from non‐small cell lung cancer (NSCLC). All investigated patients were surgically treated in the Department of Thoracic Surgery, Niels‐Stensen‐Kliniken, Ostercappeln (Germany) during December 1998 and November 2004 and had stage I to stage III tumors. We identified *n* = 379 patients. Due to tissue loss on TMAs and uncertain primary histology, tissue samples from *n* = 304 were available for evaluation. Since the patients were treated between December 1998 to November 2004, Tumor Nodule Metastasis (TNM)‐classification based on the proposed Union Internationale Contre le Cancer (UICC) sixth edition^47^ was applied.

### Immunohistochemistry

**Figure 1:**
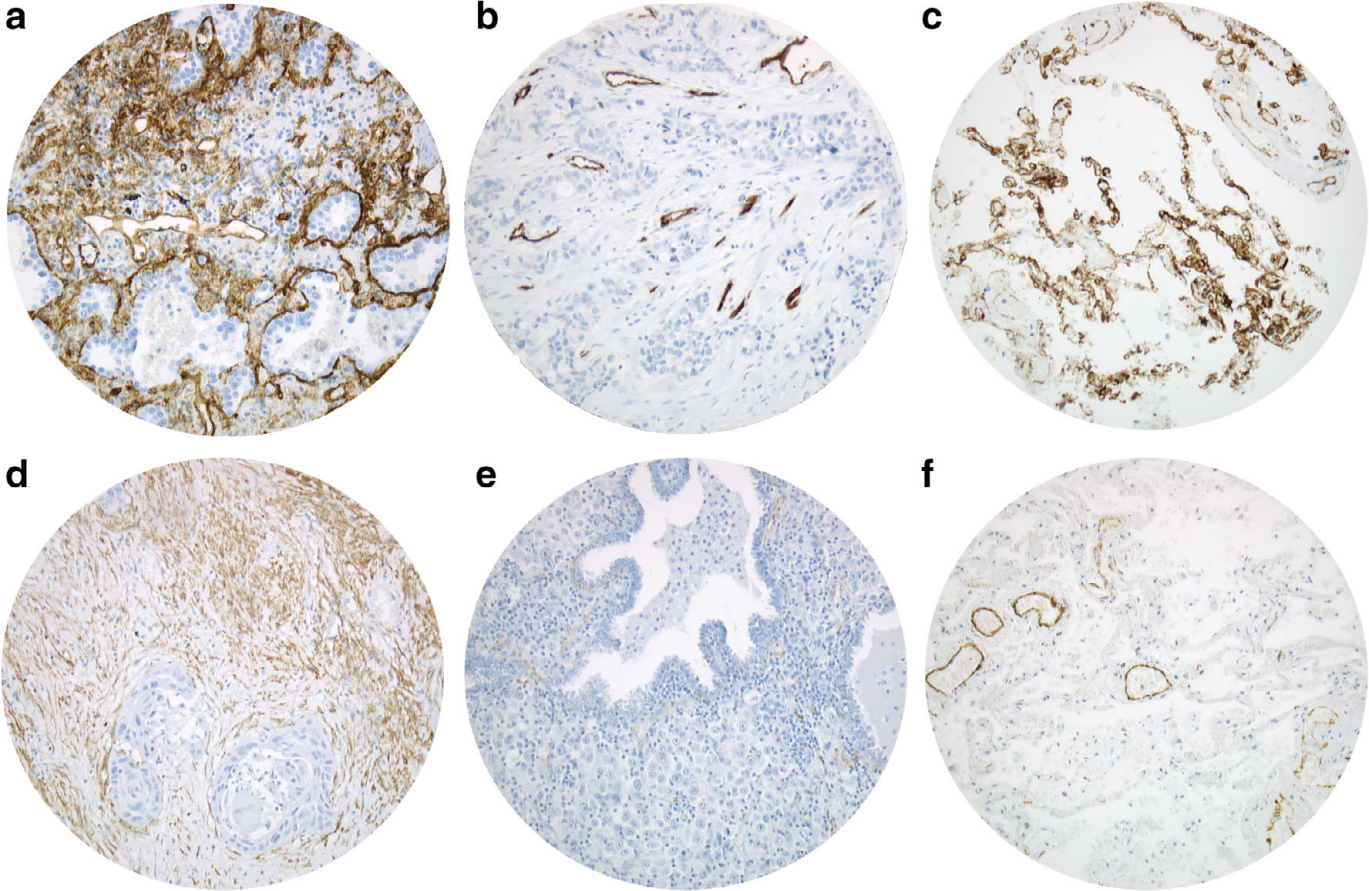
Positive and negative CD34 and SMA CAF tumor stains and healthy lung tissue stains. Upper right (**a**) positive CD34 staining, upper middle (**b**) negative CD34 staining (only vascular endothelium is stained positive), upper left (**c**) healthy lung tissue CD34 staining. Lower right (**d**) positive SMA staining, lower middle (**e**) negative SMA staining (only vascular endothelium is stained positive), lower left (**f**) healthy lung tissue SMA staining. Analyses were performed on an Olympus BX51 microscope at 200x magnification.

All surgically resected tissue samples were analyzed using 4 μm thick formalin‐fixed paraffin‐embedded (FFPE) tissue microarrays (TMA). Each NSCLC patient was represented by three punch cores.^48^ Core positions were chosen from the original hematoxylin‐eosin stained diagnostic material and transferred to the paraffin embedded tissue specimen. Each core was chosen to represent an adequate amount of representative tumor and stromal tissue. Immunohistochemistry was performed via peroxidase‐conjugated avidin‐biotin method. Primary antibodies included Roche/Ventana QBEnd/10 (CD34) and DAKO HHF35 (SMA). Briefly, after deparaffinization of TMA samples via xylene, rehydration was performed by application of graduated ethanol‐solutions at indoor temperature. Primary antibodies were incubated for 30 minutes at indoor temperature. Washing was then followed by incubation of the preparated sections with biotinylated secondary antibodies. Here, 3‐amino‐9‐ethylcarbazole was used as a substrate, and immunoreactions were detected (Ventana Optiview DAB IHC detection KIT, Germany). Healthy lung tissue served as a negative control, whereas positive controls were performed on tonsil tissue (CD34) and appendix vermicularis tissue (SMA). All samples were analyzed manually using an Olympus BX51 microscope at 200x magnification by ABS, LHS, BH and JR, respectively. The proportional amount of CD34+ and SMA+ cells from overall tumor stroma was recorded. To count CD34+ and SMA+ fibrocytes and fibroblasts, CD34+ and SMA+ endothelial cells were visually excluded from positivity in tumor stroma (*c.f*. Fig 1). Before statistical evaluation, the results were gathered and compared. Deviations were discussed interdisciplinary. PD‐1 and PD‐L1 stain in this collective were performed beforehand.^49^

### Statistical analysis

To investigate the NSCLC cohort, we used mean and standard deviation (SD), median and interquartile range (IQR; Q1–Q3), as well as raw count and frequencies. Bivariate associations between categorical variables were analyzed via Fisher's exact test or Chi‐square test, if applicable. Continuous and ordinal variables were tested using either unpaired *t*‐test or Mann‐Whitney‐U test depending on the normality of the data. Associations of more than two groups (eg, histology variable) with categorical and continuous outcomes were analyzed using Chi‐square test or Kruskal‐Wallis test. The area under the receiver operating characteristic curve (ROC) was used to determine cutoff values for CD34 and SMA‐stain via Youden Index regarding median overall survival.

Overall survival was defined as time (days) between histopathological diagnosis and death or censoring. Progression free survival was defined as time (days) between histopathological diagnosis and first relapse, progress after initial treatment, death or censoring, depending on the first chronological appearance. In univariate analyses, survival time was compared between groups using Log rank tests and visualized by Kaplan Meier plots. Multivariate survival analyses were performed via Cox proportional hazards model. Forward variable selection was applied using likelihood ratio tests. Inclusion criterion was set to 0.05. Hazard ratios (HR) are presented with 95% confidence intervals (95% CI). Data collection as well as calculations were performed using IBM SPSS Statistics Version 25 (released 2017, IBM Corp., Armonk, NY, USA). The local significance level was set to 0.05. An adjustment to multiplicity was not determined as the analysis was explorative.

## Results

Baseline characteristics of the study collective (*n* = 304 stage I–III NSCLC patients) are presented in Table 1. In brief, 80% of the patients were male and 52% had stage I disease. The predominant histopathologic subtype was squamous cell carcinoma (SCC, 48%), followed by adenocarcinoma (ADC, 37%) and large cell carcinoma (LCC, 15%). While a large proportion of the patients were smokers (78%), mean relative forced expiratory volume in one second (FEV_1_) was 81.0 (± 20) %. At a median follow‐up time of 2712 days, the survival predictor of median progression free survival (PFS) was 927 (95% CI: 686.8–1167.2) days, and median overall survival (OS) was 1316 (95% CI: 986.1–1645.9) days, respectively. Within this study collective, PD‐L1^49^ expression was found in 26% of the patients (>5% tumor cell expression).

**Table 1:** Baseline characteristics of the analyzed cohort

		n_total_ = 304	Percentage of nonmissing values
Age	Mean (± SD)	65.34 (± 8.48)	
years	Median (Q1–Q3)	65.67 (61.50–70.74)	
Sex	Male	243	79.9
	Female	61	20.1
ECOG	0	44	14.5
	I	240	78.9
	II–III	20	6.6
Smoking status (*n = 303*)*	Negative	68	22.4
	Positive	235	77.6
FEV1	≥80%	155	52.9
(*n = 293*)*	<80%	138	47.1
Histopathology	Squamous cell (SCC)	146	48.0
	Adenocarcinoma (ADC)	112	36.8
	Large cell (LCC)	46	15.1
Grade (*n = 301*)*	G1	3	1.0
	G2	94	31.2
	G3	166	55.1
	G4	38	12.6
Resection	R0	286	95.0
(*n = 302*)*	R1	13	4.3
	R2	2	0.7
PD‐L1 status	0–5%	225	74.3
(*n = 303*)*	> 5%	78	25.7
UICC6 pT	pT1	96	31.6
	pT2	167	54.9
	pT3	29	9.5
	pT4	12	3.9
UICC6 pN	pN0	180	59.2
	pN1	69	22.7
	pN2	55	18.1
UICC6 cM	cM0	304	100.0
UICC6 pTNM stage	Stage I	157	51.6
	Stage II	78	25.7
	Stage III	69	22.7
Neoadjuvant treatment	No neoadjuvant treatment	214	70.4
	chemotherapy/ radio‐chem.	90	29.6
Adjuvant treatment	No adjuvant treatment	238	78.3
	chemotherapy	13	4.3
	radiotherapy	53	17.4
CD34 stain	Negative (0% stromal cells)	182	59.9
	Positive (≥1% stromal cells)	122	40.1
SMA stain	Negative (<20% stromal cells)	201	66.1
	Positive (≥20% stromal cells)	103	33.9
Progression free survival (PFS) days	Median (95% CI)	927 (686.8–1167.2)	
Overall survival (OS) days	Median (95% CI)	1316 (986.1–1645.9)	
Follow‐up days	Median (95% CI)	2686 (2542.1–2829.9)	

FEV1, forced expiratory volume in one second; Q1–Q3, interquartile range (IQR): quartile 1 (25%) to quartile 3 (75%); 95% CI, 95% confidence interval; SD, standard deviation; missing cases are indicated by the total number of evaluated patients under the categorial variable in italics (*n =).

Upon immunohistochemical evaluation for CD34‐positivity, 40.1% of the analyzed cases demonstrated positivity (defined as ≥1% of stromal cells), visually excluding microvessels and lymphatic vessels, with a mean staining of 6.9 (± 14.4) % of the stromal tissue and 33.9% SMA‐positivity (defined as ≥20% of stromal cells), harboring a mean staining of 13.7 (± 11.70) % of the stromal cells. In total, 244 of 304 (80.2%) cases showed any stromal SMA‐positivity. An association of stromal SMA+ and CD34+ tissue was not found (*P* = 0.459). Fig 1 depicts examples of stained tumor specimen as well as healthy lung tissue.

Neither CD34+ nor SMA+ CAFs (all *P*‐values > 0.05) were associated with sex. Similar, age (<65 years vs. ≥65 years; all *P*‐values > 0.05) and smoking status (non‐smoker vs. smoker; all *P*‐values > 0.05) at initial diagnosis did not deduce to stromal CD34‐positivity or SMA‐positivity. However, stromal CD34‐positivity was preferably found in pulmonary adenocarcinoma (ADC 51.8% positivity vs. SCC 34.2% positivity vs. LCC 30.4% positivity; *P* = 0.006) and was more common in differentiated tumors than in undifferentiated ones (G1 100% positivity, G2 45.7% positivity, G3 40.4% positivity, G4 15.8% positivity; *P* = 0.002). Unlike CD34 expression, stromal SMA expression neither accumulated in a specific histologic subtype (*P* = 0.939) nor was the grade of differentiation a predictor for SMA expression (*P* = 0.431). In contrast, SMA‐positivity occurred significantly more often in higher tumor stages (stage I 28.7%, stage II 34.6%, stage III 44.9%, *P* = 0.021). Relating thereto, CD34‐positivity was not associated with TNM tumor stage (*P* = 0.990). Consequently, CD34 expression was neither associated with primary tumor size and localization (pT stage, *P* = 0.368) nor with lymphonodal spread (pN stage, *P* = 0.679). On the contrary, SMA‐positivity inversely correlated to lymphonodal spread (pN0 29.4%, pN1 39.1%, pN 2 41.8%, *P* = 0.048) but not to pT stage (*P* = 0.936).

**Figure 2:**
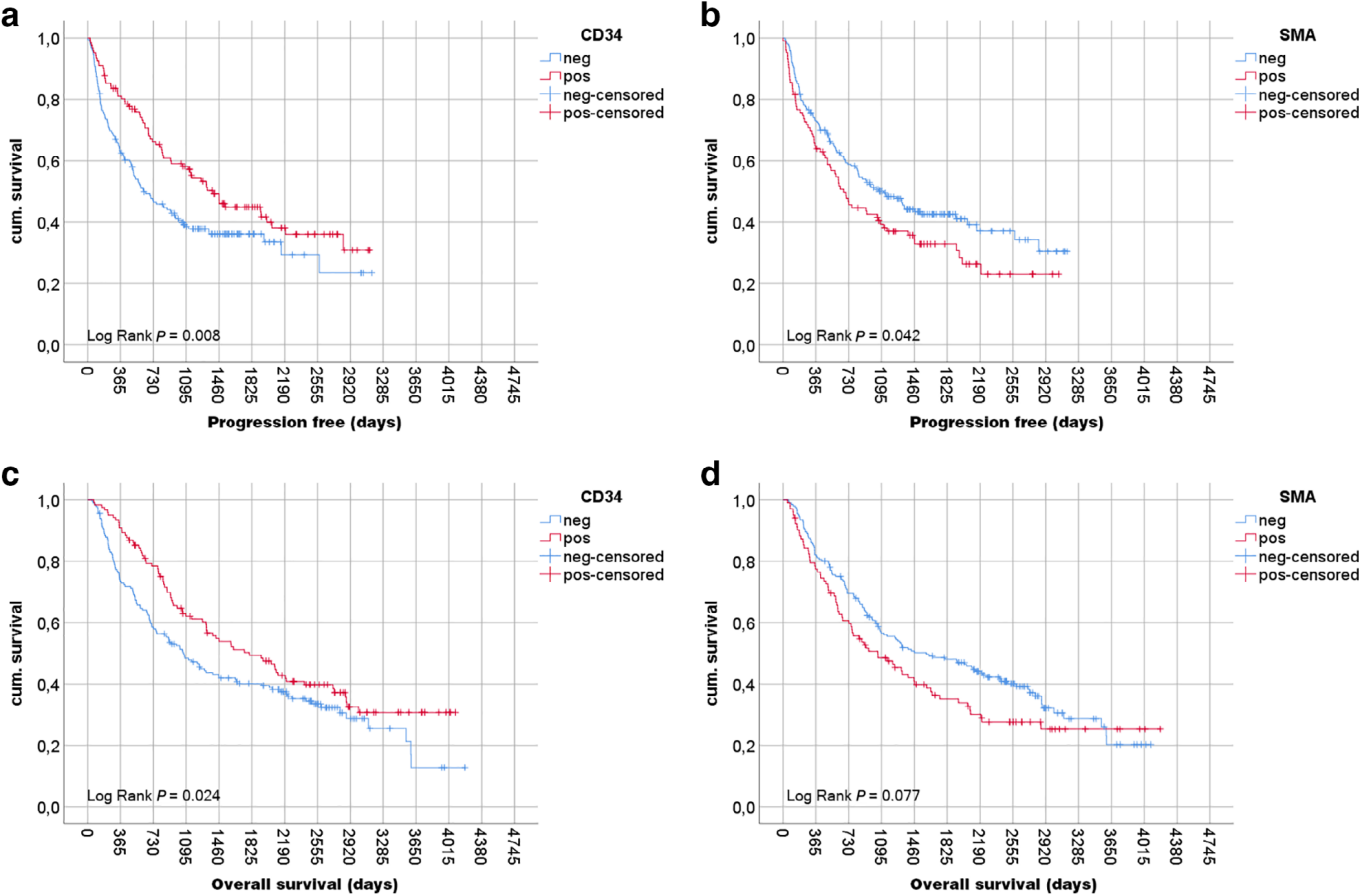
Progression free survival and overall survival depending on stromal CD34 and SMA stain. Kaplan‐Meier plots of progression free survival (PFS) and overall survival (OS) depending on stromal CD34 stain (negative = no stromal staining, positive ≥1% of nonvascular stromal cells are CD34+) and stromal SMA stain (negative <20% of stromal cells are SMA+, positive ≥20% of stromal cells are SMA+). Red curves indicate positive staining, blue curves negative tissue staining. Survival analyses were performed via Log Rank test in days since histopathological diagnosis. Vertical bars indicate oneyear survival (365 days), respectively. Analyses revealed significant survival benefits for (**a**) CD34 stained stromal tissue for PFS (*P* = 0.008) and (**b**) OS (*P* = 0.024). (**c**) SMA negativity resulted in an improved PFS (*P* = 0.042) but (**d**) not in a significant OS benefit (*P* = 0.077) in stage I–III NSCLC patients.

Figure 2 displays both PFS and OS. Here, stromal CD34‐positivity was associated with improved prognosis. While median PFS (Fig 2 **a**) was 622 (95% CI: 389.3–854.7) days for CD34 negative tissue, CD34‐positivity resulted in 1391 (95% CI: 1051.9–1730.1) days (*P* = 0.008). Similar, median OS in stromal CD34+ tumor patients (Fig 2 **c**) was 1798 (95% CI: 1226.0–2370.0) days, while CD34‐negativity resulted in reduced median survival of 1068 (95% CI: 798.7–1337.3) days (*P* = 0.024). In contrast, SMA‐negativity resulted in a PFS of 1121 (95% CI: 768.2–1473.8) days, whereas SMA‐positivity was reduced to 676 (95% CI: 511.4–840.6) days (Fig 2 **b**; *P* = 0.042). Alas, we could not prove beneficial median OS for SMA negative tumor cases (Fig 2 **d**; *P* = 0.077). Additional analyses regarding the histological subtype promoted a strong OS effect for the presence of CD34+ CAFs in LCC (*P* = 0.012). However for PFS this effect was not present (*P* = 0.228). Alas if focused on SCC or ADC solely, CD34+ CAFs did not relevantly influence PFS and OS (all *P* > 0.05, data not shown). Alike, SMA+ CAFs neither influenced PFS nor OS in ADC, SCC or LCC subcohorts (all *P* > 0.05, data not shown).

Interestingly, CD34‐positivity was particularly associated with tumors of high differentiation (PFS G1/2 *P* = 0.011 vs. G3/4 *P* = 0.187; OS G1/2 *P* = 0.027 vs. G3/4 *P* = 0.272), whereas SMA‐negativity resulted in an improved outcome in low differentiated tumors (PFS G1/2 *P* = 0.460 vs. G3/4 *P* = 0.015; OS G1/2 *P* = 0.388 vs. G3/4 *P* = 0.057) (data not shown). Consequently, Cox proportional hazards models for PFS and OS (Table 2) including TNM stage (I vs. II vs. III), age (<65 years vs. ≥65 years), sex (female vs. male), histologic pattern (SCC vs. ADC vs. LCC vs. NOS), tumor grading (G1/2 vs. G3/4), stromal SMA stain (positive vs. negative) and stromal CD34 stain (negative vs. positive) were performed, respectively. Here, among age, sex (only PFS; male HR 1.62 – *P* = 0.023), histology (only OS; ADC HR 0.71 – *P* = 0.04) and TNM stage (PFS stage II HR 1.81, stage III HR 3.37 – *P* < 0.001; OS stage II HR 1.64, stage III HR 2.97 – *P* < 0.001), stromal CD34‐negativity sustained an independent prognostic factor and harbored a hazard ratio of 1.67 (*P* = 0.001) for PFS and 1.39 (*P* = 0.028) for OS, when compared to stromal CD34+ tumors. However, tumor grading or stromal SMA expression was not identified as an independent prognostic factor in PFS or OS, respectively.

**Table 2:** Cox proportional hazards model

		Progression free survival		Overall survival	
		HR (95% CI)	*P*‐value	HR (95% CI)	*P*‐value
Age	< 65 years†		0.028		0.003
	≥ 65 years	1.40 (1.04–1.89)		1.55 (1.16–2.08)	
Sex	Female†		0.023		*0.100*
	Male	1.62 (1.07–2.46)		—	
Histology	Squamous cell carcinoma (SCC)‡		*0.075*		0.017
	Adenocarcinoma (ADC)	—	*0.186*	0.71 (0.51–0.98)	0.040
	Large cell carcinoma (LCC)	—	*0.033*	*1.29 (0.87–1.92)*	*0.201*
UICC 6 TNM stage	I‡		< 0.001		<0.001
	II	1.81 (1.26–2.60)	0.001	1.64 (1.17–2.32)	0.005
	III	3.37 (2.35–4.82)	< 0.001	2.97 (2.10–4.20)	<0.001
Grade	G1/2†		*0.060*		*0.104*
	G3/4	—		—	
Stromal SMA stain	Positive†		*0.927*		*0.980*
	Negative	—		—	
Stromal CD34 stain	Negative†		0.001		0.028
	Positive	1.67 (1.22–2.28)		1.39 (1.04–1.88)	

Cox proportional hazards model using a forward likelihood ratio test; inclusion criterion 0.05.

Italic values are not significant.

† Index variable.

‡ Reference variable for type III test.

To rule out the possibility that neoadjuvant treatment might have altered stromal CD34 and SMA levels we performed correlative analyses via contingency tables and Fisher's exact test. Neither CD34‐positivity nor SMA‐positivity was associated with an applied neoadjuvant treatment regimen (*P* = 0.308 and *P* = 0.147). Yet, positive PD‐L1 expression was associated with stromal SMA‐negativity (*P* = 0.008) but not with CD34‐positivity (*P* = 0.894). Although there was a significant association with tumor stage (*P* < 0.001), we focused on the impact of adjuvant therapies in the light of CD34‐positive CAFs and SMA‐positive CAFs in stromal tumor tissue. Here, only surgicallytreated patients revealed SMA‐dependent outcomes in PFS (*P* = 0.038) and OS (*P* = 0.038), whereas for the smaller subgroup of *n* = 66 adjuvant treated patients statistical significance could not be shown (PFS and OS both *P* > 0.05, data not shown). For cases with CD34+ CAFs, PFS was superior in pure surgically treated patients (*P* = 0.05) and adjuvant treated patients (*P* = 0.048). However, these data could not be reproduced for OS (both *P* > 0.05).

## Discussion

Cytotoxic chemotherapies are directed towards rapidly dividing cells and therefore primarily cause damage to malignant tissue. In solid tumors the microenvironment plays a crucial role in cancer development and progression. Consequently, targeted therapies against the microenvironment might enhance therapeutic efficacy.^50^ Here, we analyzed the effect of CD34+ and SMA+ CAFs on prognosis in NSCLC.

CD34 expression is a marker for fibrocytes and fibroblasts as well as neovascular endothelial cells, accounting for the evaluation of tumors' microvessel density (MVD). Evaluating MVD, high CD34+ levels are associated with greater tumor vascularization, larger tumors and a higher proportion of lymphonodal and distant metastatic spread.^35^, ^46^, ^51^, ^52^ In contrast, high CD34 levels in EMT‐partaking fibroblasts and fibrocytes were considered to be predictors of healthy tissue and benign tissue alterations in pancreatic tissue,^26^ head‐ and neck mucosa tissue,^45^ ductal gland tissue of the breast^44^ as well as tissue of the uterine cervix.^27^ In particular, the loss of CD34+ fibrocytes was associated with malignancy.^26^, ^27^, ^44^ Interestingly, in the present study we were able to correlate the grade of dedifferentiation of malignant NSCLC tissue to the proportional appearance of CD34+ fibroblasts. Whereas low graded tumors (G1) were all CD34+, lower stromal CD34+ fibroblast expression correlated with higher grading (G3 40.4% and G4 15.8% positivity, respectively). Moreover, stromal CD34+ CAF count was significantly associated with PFS and OS independently of age, sex, TNM‐stage, histology, or tumor grading in stage I–III NSCLC patients. Furthermore, CD34+ CAFs occurred more often in pulmonary adenocarcinoma (ADC) than in squamous‐cell carcinoma (SCC) or large cell carcinoma (LCC) of the lung (*P* = 0.006). However, tumor grading was significantly lower for ADC than for SCC or LCC (*P* < 0.001). In this context, further investigations are needed to determine if the predominant histological growth pattern or the degree of dedifferentiation is the major cause of altered stromal CD34+ fibroblast levels.

SMA+ CAFs have already been identified as a predictor for malignancy and higher tumor stages in various tumor entities such as pancreas,^26^ head‐ and neck squamous cell carcinoma,^45^ ductal adenocarcinoma of the breast,^44^ colon,^31^ cholangiocarcinoma^30^ or squamous cell NSCLC.^46^ Interestingly, the latter two studies reported high levels of SMA+ CAFs in both the primary tumor and lymphonodal macrometastases, whereas SMA levels showed low expression in lymphonodal micrometastases.^30^, ^46^ In our study, only primary tumor's tissue was evaluated towards the CAF infiltration, whereas further analyses on lymph node tissue were not performed. Yet, we were able to demonstrate, that in the primary lesion of the tumor, SMA+ CAFs were associated with higher TNM stages (stage I 29%, stage III 44% positivity) and lymphonodal spread (pN0 29%, pN2 42% positivity). However, in our analysis the absence of SMA+ CAFs positively influenced PFS of NSCLC patients, but not OS, and hence was not found to be an independent prognostic marker. Interestingly, tumoral positive PD‐L1 stain^49^ was associated with SMA+ CAFs. Further investigations are needed to understand the possible immune‐modulatory effect^26^ of SMA+ CAFs on tumor cells.

While the cohort size of *n* = 304 cases is relatively large, the present study shows limitations. On the one hand, the cohort was sampled 15 years ago, resulting in a partly outdated TNM staging system. Current data security measures and ethical standards prevent an update towards the current eighth IASLC TNM staging system. Furthermore, useful data on EGFR‐, KRAS‐, ALK‐ and ROS‐mutational status of the adenocarcinoma subcohort is not present and fresh frozen tissue is no more attainable. Therefore tumor mutational analysis cannot be subsequently performed. On the other hand, our analysis provides insight into patients treated before the era of antiangiogenic treatment, specific mutation targeting or even immune‐checkpoint inhibition. Despite the awareness, that neoadjuvant treatment might change tumor biology and behavior, the exclusion of those cases would have led to limited explanatory power, especially regarding the associations of SMA+ CAFs and TNM‐ as well as pN‐stage. Hence, contingency tables were used to show an independence of stromal cell expression patterns from a therapy induced before surgical treatment and need careful consideration and validation.

It has previously been shown that CD34+ fibroblasts are preferentially expressed in healthy tissue and benign tissue alterations,^26^, ^28^, ^44^, ^45^ unlike SMA+ fibroblasts, that were associated with a higher probability of malignant venous infiltration in the study by Nishishita *et al*.^31^ and showed higher presence in primary tumors and macrometastases.^30^, ^46^ Here, we were able to demonstrate that the supposed grade of invasiveness,^45^ characterized by the TNM tumor stage and its lymphonodal spread, is related to the level of SMA+ CAFs in stage I‐III NSCLC. As already mentioned, these findings need further investigation in stage IV NSCLC and stage II‐III tissue without neoadjuvant applied systemic or local treatment measure. Moreover, our study revealed that CD34+ fibroblasts are not exclusively present in healthy tissue and benign tissue alterations, but may also occur in malignant NSCLC tissue. Yet, CD34+ CAFs are inversely associated with G3/4 differentiated tumors. When focusing on the independent predictive effect of CD34+ CAFs in stage I‐III NSCLC, whether CAFs influence EMT along with lymphatic and metastatic spread, as proposed in vitro by Kim *et al*.^25^ should be investigated, In summary, the findings are consistent with available data for CAFs in malignant tissues^26^, ^27^, ^28^, ^30^, ^31^, ^44^, ^45^, ^46^ and contribute to our understanding of CAFs in NSCLC prognosis.

## Disclosure

The authors have no conflict of interest to declare.
